# Social interaction trajectories and all-cause mortality in older adults: the Otassha study

**DOI:** 10.3389/fpubh.2023.1248462

**Published:** 2023-08-22

**Authors:** Hisashi Kawai, Manami Ejiri, Kumiko Ito, Yoshinori Fujiwara, Kazushige Ihara, Hirohiko Hirano, Hiroyuki Sasai, Hunkyung Kim, Shuichi Obuchi

**Affiliations:** ^1^Tokyo Metropolitan Institute for Geriatrics and Gerontology, Tokyo, Japan; ^2^Faculty of Medicine, Hirosaki University, Aomori, Japan

**Keywords:** all-cause mortality, community-dwelling older adults, group-based trajectory modeling, social interaction, social isolation

## Abstract

**Introduction:**

This longitudinal study aimed to identify aging trajectory patterns of social interaction by sex and determine the association between these patterns and all-cause mortality.

**Methods:**

Participants were 4,065 community-dwelling older adults (1849 men) in Japan, aged 65–89 years, who responded twice or more to a mail survey conducted between 2012 and 2020. Social interaction was examined through the frequency of face-to-face and non-face-to-face contact with non-resident family and friends. The aging trajectories of the social interaction scores were identified using group-based trajectory modeling.

**Results:**

Two groups were identified among both men and women. Among men with high-frequency interaction, a rapid decrease in the frequency of social interaction was observed after 80 years of age. Conversely, among women, the frequency tended to remain the same, even after 80 years of age. The social interaction score among those aged 65 years in the low-frequency group was approximately 4 points for men and 6 points for women. Among men, no decrease was observed; however, it tended to decline after 85 years of age among women. Among men, the factors associated with the low-frequency group were instrumental activities of daily living score, perceived financial status, and social participation, while among women, they were self-rated health and social participation. The adjusted hazard ratio in the low-frequency group for all-cause mortality was 1.72 (95% confidence interval, 1.27–1.72) for men and 1.45 (95% confidence interval, 0.98–2.14) for women.

**Discussion:**

In the low-frequency group, men had a higher risk of all-cause mortality than women. Daily social interaction from mid-age is important to reduce the risk of social isolation and all-cause mortality in later life.

## 1. Introduction

Maintaining social interaction with others by meeting and talking to them is important as it helps improve health status. Social isolation is assessed using various measurements such as the number and frequency of social interaction, the social support from others, and the range of contact persons (1); one aspect of social isolation considers it a state of deteriorated social interaction (2). Social isolation is associated with the incidence of cardiovascular diseases (3), dementia (4), long-term care (5), and mortality (6, 7). Thus, maintaining social interaction is crucial for healthy longevity.

Objective measures of social isolation include the number of people with whom a person interacts (8) and the frequency of interaction (9). These two factors decline with an increase in age (8, 9), suggesting that social interaction declines gradually with age, leading to isolation. This could result from decreased mobility because of physical deterioration (10), decreased cognitive function (11), and age-based decreased social participation (12). Further, poor self-rated health and decreased social participation are risk factors for social isolation (1). Deterioration of health status and reduced opportunities for social participation based on age may accelerate the decline in the frequency of social interaction and the incidence of social isolation. However, the trajectory of the decline in the frequency of social interaction is not fully understood.

Studies have identified aging trajectories using group-based trajectory modeling for longitudinal data (13–18). This method can classify the patterns of an outcome’s evolution over age or time from fragmental data (19). Regarding physical performance and higher-level functional capacity in older individuals, several trajectory patterns, such as a pattern with gradual decrease with age, a pattern with rapid decrease, and a pattern with a decrease during the early phase, were observed (17, 18). Such age-related declines in physical function may rapidly decrease social interaction. One study showed that social network size declined with age (20), especially in later life; however, it did not clarify the patterns of the decline of social network size with aging. Additionally, a previous study reported sex-related differences in the association between social isolation and functional decline (21); however, sex-related differences in the trajectories of social interaction are unclear. To our knowledge, no studies have identified the aging trajectory patterns of social interaction by sex. A recent systematic review assessing the factors of social isolation revealed that compared to women, men have higher risk for social isolation (1), suggesting that reduced social interaction is also likely to be more pronounced in men. Identifying these trajectories can help determine how social interaction decreases with age and leads to isolation. Examining the factors associated with such trajectories and their impact on health outcomes can provide useful insights into preventive measures against social isolation.

Therefore, this study aimed to identify the aging trajectory of social interaction frequency by sex—a measure of social isolation—using longitudinal data from a cohort of community-dwelling Japanese older adults. Furthermore, the factors associated with trajectory patterns and the relationship between such patterns and all-cause mortality were examined. In this study, we hypothesize that the aging trajectory patterns of social interaction frequency differ by sex, including patterns with rapid decrease such as physical function.

## 2. Materials and methods

### 2.1. Participants

Data were obtained through a mail survey conducted between 2012 and 2020 from the Otassha Study. In the cohort study, participants were sampled from over 7,000 residents aged 65–85 years who lived in nine areas near Itabashi-ward, Tokyo, Japan, excluding institutionalized residents and participants of previous surveys conducted by our institute. Inclusion and exclusion criteria were residents described above who responded to the survey and did not respond, respectively. Details of this cohort are described elsewhere (22).

In the 2012 first mail survey (Wave 1), a questionnaire was sent to 7,015 older residents. Of these, 3,696 responded (response rate: 52.7%). In 2013 and 2014, new participants aged 65 years were added, and second and third mail surveys (Waves 2 and 3) were conducted. Although follow-up surveys were not conducted in 2015 and 2016 due to limited research funds, annual surveys were conducted with the past respondents and new participants aged 65 years from 2017 to 2020 (Waves 4–7; Table 1). The inclusion criterion for the present study was participants who completed the items on social interaction at least twice during the seven surveys. Finally, 81.8% of the first mail survey participants were included in the analysis of this study. Although there was no significant difference in the men to women ratio between participants and dropouts, the age of the dropouts (74.5 years) was significantly higher than that of the participants (72.7 years). Other missing data were handled by the group-based semiparametric mixture modeling.

**Table 1:** Time, participants, and response rate of each mail survey.

Wave	Wave 1	Wave 2	Wave 3	Wave 4	Wave 5	Wave 6	Wave 7
Time (year)	2012	2013	2014	2017	2018	2019	2020
Participants	Residents aged 65–85 who lived in survey areas	Residents aged 65–86 who lived in survey areas	Residents aged 65–87 who lived in survey areas	Past respondents and new participants aged 65 years	Past respondents and new participants aged 65 years	Past respondents and new participants aged 65 years	Past respondents and new participants aged 65 years
Sent (*n*)	7,015	7,128	7,737	4,215	4,267	3,745	3,289
Response (*n*)	3,696	3,656	3,522	2,738	2,594	2,395	2,118
Response rate (%)	52.7	51.3	45.5	65.0	60.8	64.0	64.4

All respondents of the mail survey provided written informed consent to use their data for this study. Ethical approval for this study was granted by the ethics committee of the Tokyo Metropolitan Institute of Gerontology (no. R21-033). The study was conducted following the guidelines of the World Medical Association Declaration of Helsinki.

### 2.2. Social interaction score

Social interaction was assessed by asking participants about the frequency of face-to-face or non-face-to-face contact with non-resident family and friends (5, 9). The following four questions were asked: (i) “How often did you see your family members or relatives who are living apart?” (ii) “How often did you make contact with your family members or relatives who are living apart by phone, email, or facsimile?” (iii) “How often did you see your friends or neighbors?” (iv) “How often did you make contact with your friends or neighbors by phone, email, or facsimile?” In the 2014 and 2017 surveys, participants were asked, “How often did you see or make contact with your family members or relatives who are living apart by phone, email, or facsimile?” Owing to limited survey items, we could not differentiate between face-to-face and non-face-to-face contact. The frequency of interaction was classified as follows: (i) 6–7 times a week (almost every day); (ii) 4–5 times a week; (iii) 2–3 times a week; (iv) once a week; (v) 2–3 times a month; (vi) once a month; (vii) less than once a month; (viii) none (scored as 0 [none] to 7 [almost every day]). The total score of face-to-face or non-face-to-face contact with non-resident family members and friends was used as the social interaction score (score range: 0–14). This social interaction score was defined originally in this study; however, the items of the social interaction measure were common to those used in previous studies (5, 9, 22). Since individuals in these previous studies and several other studies (22–24) were considered to be socially isolated if they made contact less than once per week, we believed that it is reasonable to set the corresponding 4 points as the cutoff for social isolation.

### 2.3. Mortality

Information regarding mortality was obtained from October 1, 2012, to November 1, 2020, from the database administrated by the ward office. This mortality information was informed by the notification of death forms for residents.

### 2.4. Covariates

Age, sex, chronic diseases (i.e., hypertension, diabetes mellitus, stroke, and heart disease), instrumental activities of daily living (IADL), self-rated health, perceived financial status, living alone status, and social participation are associated with social isolation (1). In the current study, these factors were included as covariates in the analyses. We used covariate data from the year in which each participant first responded to the survey.

Age and IADL were entered as continuous variables and others as categorical variables. For chronic diseases, the presence or absence of each disease was entered individually. The question regarding self-rated health provided four choices: very healthy, healthy enough, not very healthy, and not healthy. The IADL score was assessed using a subscale of the Tokyo Metropolitan Institute of Gerontology Index of Competence, which includes five questions on instrumental self-maintenance (25). The question regarding perceived financial status was evaluated using five options: very comfortable, slightly comfortable, neither comfortable nor hard, a little hard, and very hard.

Social participation was examined in five activity groups: neighborhood associations, senior citizen clubs, hobby groups, sports groups, and volunteer groups (26).

### 2.5. Statistical analyses

Participants’ baseline characteristics are presented as means and standard deviations (SDs) for continuous variables and as percentages for categorical variables. The aging trajectories of the social interaction score were identified by group-based semiparametric mixture modeling (19) using TRAJ in STATA 15.1 (StataCorp LLC; College Station, Texas, United States). We used a censored normal (cnorm) model to identify the aging trajectories. The number of groups was examined for 2, 3, and 4 with cubic curve models; the best-fitting model was selected by comparing the Bayesian information criterion values. Sex-stratified analysis was also conducted for the aging trajectories of the social interaction score.

To identify factors associated with the aging trajectory patterns of social interaction, multiple logistic regression analyses were conducted. The dependent variable was the trajectory patterns, and explanatory variables were the baseline data on sex, age, self-rated health, living alone status, perceived financial status, chronic disease, social participation, and IADL score.

The association between aging trajectory patterns of social interaction and all-cause mortality was investigated using Cox proportional hazards models, which started from the baseline survey for each participant until death or the end of follow-up (November 1, 2020), while adjusting for sex, age, chronic disease, living alone, and IADL score. The assumption that the proportional hazards remain constant over time was assessed based on the graphs on the cumulative survival curve. All statistical analyses, except for group-based trajectory modeling, were performed using SPSS version 27 (IBM Japan, Ltd.; Tokyo, Japan).

## 3. Results

This study analyzed data from 4,065 participants (2,216 women and 1849 men) with a mean age (SD) of 71.6 (±5.6) years (Table 2). The average duration of follow-up (SD) was 5.1 (±2.9) years, the average number of follow-up assessments was 4.2 (±1.9), and the total number of observations was 17,150. The average social interaction score (SD) was 7.5 (±3.5).

**Table 2:** Participants’ baseline characteristics.

Variable	Men (*n* = 1849)		Women (*n* = 2,216)		All participants (*N* = 4,065)	
	Mean	SD	Mean	SD	Mean	SD
Age (years)	71.7	5.6	71.6	5.6	71.6	5.6
**Chronic disease (%)**
Hypertension	42.0		38.7		40.2	
Diabetes mellitus	17.2		8.3		12.4	
Stroke	6.4		2.0		4.0	
Heart disease	16.1		7.9		11.7	
IADL score	4.7	0.8	4.9	0.6	4.8	0.7
Social interaction score	6.6	3.5	8.4	3.3	7.5	3.5
**Self-rated health (%)**
Very healthy	11.3		11.2		11.2	
Healthy enough	68.3		69.4		68.9	
Not very healthy	15.3		14.8		15.0	
Not healthy	5.1		4.7		4.9	
**Perceived financial status (%)**
Very comfortable	3.2		3.8		3.5	
A little comfortable	32.3		34.9		33.7	
Neither comfortable nor hard	39.1		41.6		40.4	
A little hard	20.7		16.0		18.1	
Very hard	4.7		3.8		4.2	
Living alone (%)	16.3		24.7		20.9	
**Social participation (%)**
Neighborhood associations	26.2		26.9		26.6	
Senior citizen clubs	8.3		11.9		10.3	
Hobby groups	22.9		39.1		31.8	
Sports groups	18.7		26.2		22.8	
Volunteer groups	7.0		8.6		7.9	
Duration of follow-up (years)	4.9	2.9	5.3	2.8	5.1	2.9

SD, standard deviation; IADL, instrumental activities of daily living.

**Figure 1:**
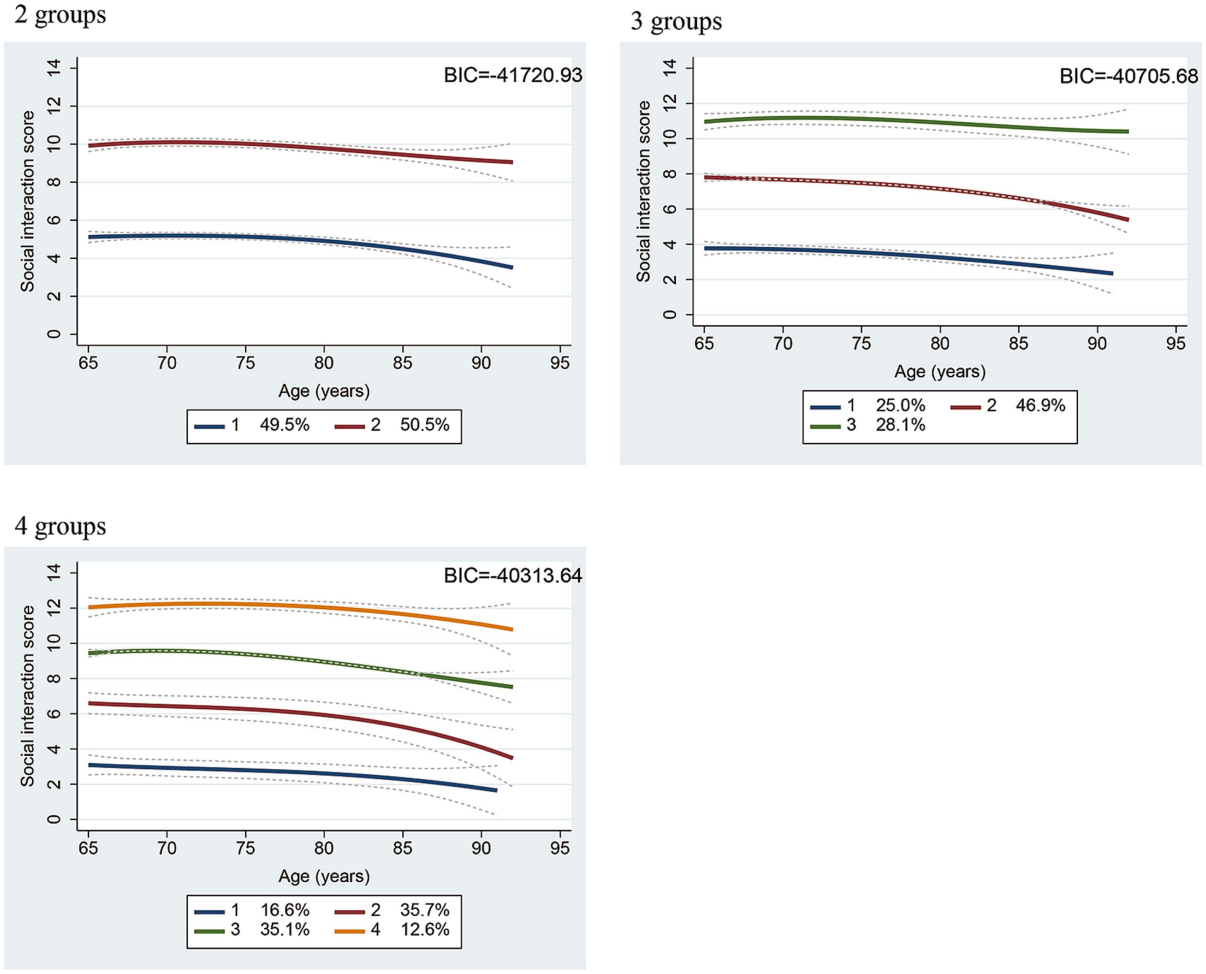
Two, three, and four trajectories for social interaction scores identified by a group-based semiparametric mixture model among all participants. BIC, Bayesian information criterion; Dotted line: 95% confidence interval.

For the aging trajectories of social interaction scores, because two to four group models were examined, the two-group cubic trajectory model was identified as the best-fitting model based on the Bayesian information criterion values (Figure 1). Two trajectory patterns were observed: high-frequency and low-frequency groups (Figure 1). In the high-frequency group, the social interaction score for 65 years of age was approximately 10 points, and high-frequency contact almost every day was maintained until approximately 80 years of age. In contrast, in the low-frequency group, the social interaction score for 65 years of age was approximately 5 points, with a gradual decline in contact frequency. The score settled below 4 points, reaching the level of social isolation at approximately 80 years of age.

**Figure 2:**
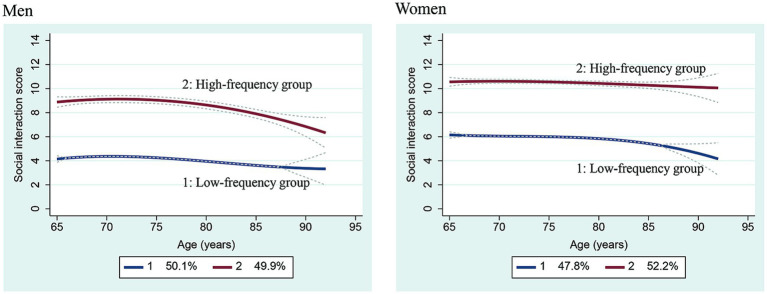
Two group trajectories for social interaction scores among men and women. Dotted line: 95% confidence interval.

Similarly, two groups were identified by sex in a stratified analysis (Figure 2). Among men with high-frequency interaction, a rapid decrease in the frequency of social interaction was observed after 80 years of age. Conversely, among women, the frequency tended to remain the same, even after 80 years of age. The social interaction score among those aged 65 years in the low-frequency group was approximately 4 points for men and 6 points for women. Among men, no decrease was observed; however, it tended to decline after 85 years of age among women.

Factors associated with the low-frequency group included male sex, younger age, no history of stroke, poor IADL score, poor self-rated health, poor perceived financial status, and poor social participation (Table 3). A stratified analysis by sex showed that the IADL score, perceived financial status, and social participation were associated with the trajectories of social interaction among men. Factors associated with such trajectories among women included hypertension, self-rated health, and social participation.

**Table 3:** Factors associated with the trajectory patterns for social interaction scores.

	Men		Women		All participants	
	OR	95% CI	OR	95% CI	OR	95% CI
Sex (reference: women)	–	–	–	–	**2.44**	2.10–2.82
Age (years) (1-year increments)	1.01	0.99–1.03	**0.98**	0.96–0.99	**0.98**	0.97–0.99
**Chronic disease (reference: none)**
Hypertension	0.90	0.73–1.11	**0.81**	0.66–0.99	0.93	0.80–1.08
Diabetes mellitus	1.00	0.75–1.32	0.85	0.60–1.21	0.90	0.72–1.13
Stroke	0.74	0.47–1.17	0.74	0.36–1.52	**0.67**	0.46–0.97
Heart disease	1.18	0.89–1.58	0.76	0.53–1.10	1.07	0.85–1.34
IADL score (1-point increments)	**0.78**	0.67–0.92	0.86	0.71–1.04	**0.86**	0.77–0.97
**Self-rated health (reference: very healthy)**
Healthy enough	1.40	0.99–1.98	**1.97**	1.41–2.75	**1.68**	1.32–2.14
Not very healthy	1.50	0.97–2.32	**2.55**	1.69–3.85	**2.17**	1.60–2.94
Not healthy	1.83	0.98–3.41	**4.67**	2.49–8.78	**2.74**	1.76–4.26
**Perceived financial status (reference: very comfortable)**
A little comfortable	1.55	0.81–2.95	1.12	0.67–1.89	1.14	0.76–1.72
Neither comfortable nor hard	**2.08**	1.09–3.95	1.29	0.77–2.17	**1.51**	1.00–2.26
A little hard	**2.09**	1.08–4.06	1.30	0.75–2.25	**1.61**	1.05–2.46
Very hard	**3.40**	1.49–7.75	1.65	0.80–3.41	**2.36**	1.36–4.08
Living alone (reference: not living alone)	1.30	0.97–1.74	0.84	0.68–1.05	0.99	0.82–1.18
**Social participation (reference: participation)**
Neighborhood associations	**1.88**	1.47–2.41	**1.37**	1.10–1.72	**1.68**	1.42–1.99
Senior citizen clubs	1.49	0.99–2.25	0.91	0.66–1.25	1.08	0.84–1.40
Hobby groups	**2.09**	1.61–2.72	**1.81**	1.47–2.22	**1.71**	1.45–2.01
Sports groups	**2.02**	1.52–2.69	**1.50**	1.20–1.88	**1.57**	1.31–1.87
Volunteer groups	1.21	0.79–1.86	1.24	0.87–1.78	**1.57**	1.18–2.09

CI, confidence interval; OR, odds ratio; IADL, instrumental activities of daily living; numbers in bold are statistically significant; dependent variable: pattern of trajectories for social interaction score (reference: high-frequency group).

During the follow-up period, all-cause deaths occurred in 125 (6.0%) and 198 (9.9%) participants in the high- and low-frequency groups, respectively. The adjusted hazard ratio (HR) for all-cause mortality in the low-frequency group was 1.48 (95% confidence interval (CI): 1.16–1.88; Table 4). The stratified analysis by sex revealed a significant association and the adjusted HR was 1.72 (95% CI, 1.27–2.32) in men. The adjusted HR was 1.45 (95% CI, 0.98–2.14) in women, which was not significant.

**Table 4:** Independent associations of social interaction trajectories with all-cause mortality.

Social interaction trajectory	Incident all-cause deaths		All-cause mortality	
			Crude	Adjusted
	*n*	%	HR (95% CI)	HR (95% CI)
**All participants**
High-frequency group	125	6.0	1	1
(*n* = 2070; 50.9%)				
Low-frequency group	198	9.9	1.72	1.48
(*n* = 1995; 49.1%)			(1.37–2.15)	(1.16–1.88)
**Men**
High-frequency group	72	7.9	1	1
(*n* = 915; 49.5%)				
Low-frequency group	131	14.0	1.89	1.72
(*n* = 934; 50.5%)			(1.42–2.52)	(1.27–2.32)
**Women**
High-frequency group	52	4.4	1	1
(*n* = 1,174; 53.0%)				
Low-frequency group	68	6.5	1.52	1.45
(*n* = 1,042; 47.0%)			(1.06–2.19)	(0.98–2.14)

CI, confidence interval; HR, hazard ratio; numbers in bold are statistically significant; Cox hazards regression models adjusted for sex, age, chronic diseases, instrumental activities of daily living score, and living alone.

## 4. Discussion

The present study identified the aging trajectories of social interaction scores using large-scale longitudinal data from a cohort of community-dwelling older adults and classified them into two groups. Overall, social interaction scores did not change rapidly with aging, even when divided into 3 or 4 groups. The high-frequency group showed a pattern where the social interaction score was 7 points or more, and interaction with others almost every day was maintained until over 80 years of age. Conversely, the low-frequency group had a score of approximately 5 points, indicating that they interacted with others 2–3 times a week at 65 years of age. The score dropped to less than 4 points at approximately 80 years of age, indicating that interaction occurred once a week. This study had over 4,000 participants, with an average follow-up duration of approximately 5 years, and it included more participants and a similar follow-up duration as compared to a previous study on aging trajectories (18). Therefore, the trajectories of the social interaction scores identified in this study may better represent the trajectories of community-dwelling Japanese older adults.

Limited studies have reported the aging trajectories of variables concerning social interaction in older adults. These studies analyzed the aging trajectories of basic activities for daily living, physical performance such as grip strength and walking speed (17), and higher-level functional capacity (18) and reported patterns where function declined rapidly with age. Thus, we deduced that social interaction also followed patterns in which interaction declined rapidly. However, those patterns were not found in the present study, suggesting that individuals exposed to an increased frequency of social interaction can maintain the level of social interaction, even if their physical function declines. The present study found that people have stable levels of social interaction throughout their later life. In a longitudinal study on age-related changes in social network size, social network size was maintained from 57 to 73 years of age (20). Therefore, to prevent social isolation, measures to ensure high-frequency social interaction are needed from a young age. It is important to promote social interaction from a young age, not just in old age. Social interaction should continue to be encouraged even as health, lifestyle, and work environment change with age. It is necessary to disseminate this information and provide opportunities for maintaining social interaction.

The sex-stratified analysis showed that the frequency of social interaction was maintained among the high-frequency group of women, even at a later age. Conversely, a gradual decline was observed among men over 75 years, followed by a rapid decline after the age of 80. The social interaction score among women aged 65 years in the low-frequency group was 6 points, which corresponds to an interaction frequency of 4–5 times a week; however, after 85 years of age, it declined to 4 points, which corresponds to possible social isolation. The score was 4 points for men, which was deemed as indicative of social isolation (5). As there are sex-related differences in social participation (27) and the association between social isolation and functional decline (21), sex-related differences in functional status and communication style could affect social interaction in later life. This study found sex-related differences in the trajectory of social interaction frequency leading to isolation. Recent studies reported that the coronavirus disease pandemic significantly increased social isolation among older adults (28–30); particularly, men aged ≥50 years experienced the greatest increase in the prevalence of social isolation (23). Thus, men in the low-frequency group must be screened at an early age and provided with timely interventions (e.g., when they are middle-aged). Many men may change their lifestyle and work environment owing to retirement in middle age. Therefore, it is necessary to share information about the importance of social interaction before reaching the age of 65 years. In women, decreased social interaction was seen in the low-frequency group over the age of 85 years, which could be attributed to decreased walking ability and muscle strength in older age. Encouraging exercise to maintain walking ability and muscle strength may prove effective for women in the low-frequency group.

In this study, male sex, younger age, no history of stroke, lower IADL scores, poor self-rated health, poor perceived financial status, and poor social participation were associated with the low-frequency group of trajectories. These factors are consistent with a previous study that examined factors of social isolation (1). Since the social interaction score in the low-frequency group in the present study was between 3 and 5 points, indicating an interaction frequency of 2–3 times a month to 2–3 times a week, the factors of the low-frequency group could be consistent with the previous study (1), where a social interaction frequency of less than once a week was defined as social isolation.

The factors associated with the trajectory patterns of social interaction among men and women varied. Self-rated health in women alone and perceived financial status in men alone were sex-specific factors affecting the trajectory pattern, suggesting that social interaction is restricted by women’s health and men’s financial status. Women face a higher risk of declining mobility than men (31), which could result in poor self-rated health, thus restricting social interaction. For men, work may be the main opportunity for social interaction; not working for an income may lead to reduced social interaction. There was no significant difference between the low-frequency and high-frequency groups for both men and women, suggesting that living alone was not an independent factor of social interaction. This result is in line with a previous study that showed that a poor social network rather than living alone was associated with adverse health outcomes (32). Indicating whether or not people live alone is not an issue of social isolation.

The adjusted HR to all-cause mortality in the low-frequency group of trajectories in social interaction was 1.48, similar to the random effect weighted average OR for 1.40 (95% CI: 1.06–1.86) in a meta-analysis of mortality owing to social isolation (6). Thus, the results suggest that the low-frequency group faces a similar risk of death to those associated with social isolation. A study examining the association between the co-existence of social isolation and homebound status, with a risk of all-cause mortality through a six-year follow-up in Japanese older adults (24), found that the adjusted HR to all-cause mortality in the group of social isolation coexisting with being homebound was 2.19 (95% CI: 1.04–4.63), which was higher than that in the current study. The risk of mortality further increased when the homebound group overlapped with the low-frequency group of the social interaction trajectory pattern.

Among men, the adjusted HR for all-cause mortality in the low-frequency group was 1.72, which was higher than that in the overall analysis, suggesting that men in the low-frequency group had a particularly high risk of mortality. As described above, this could be because this group of men is already socially isolated at 65 years. Meanwhile, among women, the association between the low-frequency group and mortality was not significant in the adjusted model. This study showed that in the low-frequency group, men had a higher risk of all-cause mortality than women.

This study is the first to identify the aging trajectories of social interaction scores in Japanese older adults using large-scale longitudinal data. Particularly, men in the low-frequency group are already socially isolated from 65 years and face a higher risk of mortality. This study suggests that early interventions (e.g., beginning in middle age) are important for men in the low-frequency group. The factors associated with men in this group were low IADL, low perceived financial status, and no participation in neighborhood associations, hobby groups, and sports groups. It is important to provide exercises that improve the walking ability to maintain IADL (33). A multicomponent intervention, comprising physical, social, health education, and environmental interventions, are suggested to be effective for promoting social participation (34). Providing the information and opportunity on those interventions from middle adulthood is important. In addition, since this study showed that perceived financial status in men is one of the factors of the low-frequency group, the risk of social isolation may reduce in men by providing employment opportunities.

This study has a few limitations. First, it was conducted in an urban area in Japan. The aging trajectories of social interaction in rural areas and other countries may vary; thus, the findings may not be adequately representative of these populations. Future studies in other regions are necessary. As this study involved a mail survey, marital status, physical activity, smoking, diet, and BMI could not be examined due to limited survey items. In addition, other potential confounders, such as social support from or relationships with family members and severe illness (e.g., cancer), could not be examined. Further, we uniquely defined the social interaction score to assess the degree of social interaction. However, the items used to calculate the score were the same as those used for assessing social interaction in the previous studies (5, 22). We believe that the degree of social isolation was quantitatively evaluated in this study. Moreover, trajectories associated with other social variables, such as social networks, must be identified (8). Although IADL may change over time, we did not treat it as a time-dependent covariate in the Cox hazards model since most of the participants in this study had independent IADL, and the change over time was assumed to be small. Further, we did not examine the risk associated with the cause of death because the incidence of mortality was not high. However, some studies have reported that social isolation can increase the risk of cardiovascular disease (3, 35). Future studies should explore the risk of aging trajectory patterns in social interaction with a focus on the cause of death.

## Data availability statement

The raw data supporting the conclusions of this article will be made available by the authors, without undue reservation.

## Ethics statement

The studies involving humans were approved by Ethics committee of the Tokyo Metropolitan Institute of Gerontology. The studies were conducted in accordance with the local legislation and institutional requirements. The participants provided their written informed consent to participate in this study.

## Author contributions

HKa and SO contributed to the design and concept of the study. HKa, ME, KIt, YF, KIh, HH, HKi, and SO acquired the data. HKa, ME, and KIt analyzed and interpreted the data as well as drafted the manuscript. YF, HS, and SO revised the manuscript for important intellectual content. All authors contributed to the article and approved the submitted version.

## Funding

This work was supported by Health and Labor Sciences Research Grants [H24-Choju-Ippan-002 and H25-Choju-Ippan-005] from the Ministry of Health, Labour and Welfare of Japan; the Promotion Project of Creating Industry Extending Healthy Life Expectancy from the Japanese Ministry of Economy, Trade and Industry; Research Funding for Longevity Sciences from the National Center for Geriatrics and Gerontology, Japan [grant numbers 28–30 and 29–42]; the longitudinal study grant from Tokyo Metropolitan Institute of Gerontology.

## Conflict of interest

The authors declare that the research was conducted in the absence of any commercial or financial relationships that could be construed as a potential conflict of interest.

## Publisher’s note

All claims expressed in this article are solely those of the authors and do not necessarily represent those of their affiliated organizations, or those of the publisher, the editors and the reviewers. Any product that may be evaluated in this article, or claim that may be made by its manufacturer, is not guaranteed or endorsed by the publisher.
